# Editorial: Brain-inspired intelligence: the deep integration of brain science and artificial intelligence

**DOI:** 10.3389/fncom.2025.1553207

**Published:** 2025-03-04

**Authors:** Ye Yuan, Xi Chen, Jian Liu

**Affiliations:** ^1^Institute of Machine Intelligence, University of Shanghai for Science and Technology, Shanghai, China; ^2^School of Software Engineering, East China Normal University, Shanghai, China

**Keywords:** brain-inspired intelligence, brain science, artificial intelligence, brain cortex, neural mechanism

The emergence of consciousness and biological behavior from neural activity represents one of the most profound and challenging questions in neuroscience (Bullmore and Sporns, 2009; Latora et al., 2017). As the cornerstone of understanding brain function, it also holds transformative potential for advancing the diagnosis and treatment of mental disorders and for the development of brain-inspired artificial general intelligence. The brain, composed of an extraordinary number of neurons with diverse morphologies and functions, forms a labyrinth of intricate structural and functional connections (Yuan et al., 2019). Deciphering the neural circuit principles that underpin cognitive functions remains a formidable scientific challenge. Thus far, extensive efforts have been devoted to unraveling how neural activity orchestrates the emergence of consciousness and governs behavior, and how the brain's structural architecture supports its extraordinary complexity—spanning scales from brain regions to individual neurons and synapses.

The medial prefrontal cortex (mPFC) plays a crucial role in behaviors involving working memory, such as planning and decision-making, but the complexity of its neural processes remains difficult to capture with current experimental designs. Studies using rodent and primate models, particularly in T-maze tasks, have highlighted the statistical limitations of existing methods, including the inability to fully leverage neuronal spike sequences and local field potentials (LFPs) for understanding neural synchrony and its behavioral relevance. Unlike the evolutionarily older visual cortex, which benefits from spatially organized and robust electrical signals, the mPFC lacks such spatial regularity, resulting in weaker signals and necessitating invasive and highly sensitive electrophysiological techniques that remain limited in scale. Recent advances, such as the use of dynamic time warping, offer potential for capturing neural synchrony, a key feature of mPFC function, but are constrained by the inadequacy of current datasets and tools. Future progress will require larger, higher-resolution datasets, innovative experimental approaches, and interdisciplinary integration of computational modeling to address these challenges and advance our understanding of how the mPFC supports complex cognitive and behavioral processes.

The visual cortex, one of the earliest areas of the brain to be studied, has played a key role in understanding visual processing mechanisms, particularly in the application of visual tasks in deep learning (Grill-Spector and Malach, 2004; Yuan et al., 2023). Through the study of simple and complex cells in the visual cortex, scientists have revealed how the brain processes different levels of visual information, which has driven the development of deep learning models such as convolutional neural networks (CNNs) (Górriz et al., 2023). These models, by simulating the processing methods of the visual cortex, have achieved breakthrough advancements in tasks such as image classification and object recognition. Research on the visual cortex not only provides deep insights into neuroscience but also offers valuable inspiration for the design of visual systems in artificial intelligence. Despite significant progress in artificial vision, there are still notable gaps between biological and artificial systems. First, convolutional neural networks (CNNs) reduce input data dimensionality through pooling, which contrasts with the increase in neurons and synapses seen in the brain's visual hierarchy (e.g., V1–V4). This discrepancy led Barlow to revise his redundancy reduction hypothesis into the redundancy exploitation hypothesis. Second, CNNs lack generalizability, especially beyond direct experiences, which is tied to the binding problem and consciousness. Achieving human-level generalization requires a compositional approach to AI. Finally, the brain's ability to manage learning and memory with trillions of synapses while consuming very little power remains an unresolved mystery.

Traditional computational neuroscience relies on hypothesis-driven, hand-engineered models, which struggle to capture the complexity of closed-loop perception-action systems. Conversely, goal-driven deep learning autonomously learns complex tasks, providing new insights into neurocomputational mechanisms. AngoraPy addresses challenges in training recurrent convolutional neural networks (RCNNs) on sensorimotor tasks, leveraging reinforcement learning (RL) to bypass the need for costly labeled data. It supports customizable architectures and tasks, enabling efficient, large-scale training with anthropomorphic sensory inputs and motor outputs. Benchmarks showcase its flexibility across diverse robotic and control tasks, making it a valuable tool for advancing sensorimotor neuroscience.

A deeper understanding of neural mechanisms has significantly advanced the development of brain-inspired artificial intelligence technologies. For example, by integrating neuroscience insights, the researchers developed a spatial cognition model inspired by the hippocampus's structure and function, particularly the cornu ammonis (CA) subregions. Leveraging brain reference architecture (BRA)-driven development, the model combines Monte Carlo localization (MCL) for allocentric mapping and a recurrent state-space model (RSSM) for learning egocentric state representations from sensory inputs. Simulations demonstrated improved self-localization during teleportation, with sparse neural activity in CA3-mimicking latent variables supporting adaptability. This approach highlights the potential of brain-inspired architectures to enhance robotic self-localization, emphasizing structural-functional consistency in adapting to unanticipated changes.

It is foreseeable that the development of neuroscience and artificial intelligence will become increasingly intertwined, with mutual promotion gradually becoming the main theme of brain science development both now and in the future.
